# Impact of solid fuel usage on respiratory symptoms among reproductive aged women: a cross-sectional study in Sri Lanka

**DOI:** 10.1186/s12889-022-14748-8

**Published:** 2022-12-03

**Authors:** Malshani Lakshika Pathirathna, Chandraratne Mahinda Bandara Dematawewa, Kayako Sekijima, Mieko Sadakata, Yoshiyuki Muramatsu, Naoshi Fujiwara

**Affiliations:** 1 Department of Nursing, Faculty of Allied Health Sciences, University of Peradeniya, Peradeniya, 20400 Sri Lanka; 2 Department of Animal Science, Faculty of Agriculture, University of Peradeniya, Peradeniya, 20400 Sri Lanka; 3 Graduate School of Health Sciences, Niigata University, 2-746, Asahimachi-Dori, Chuo-Ku, Niigata, 951-8518 Japan

**Keywords:** Biomass fuel, Air pollutant, Women’s health, Respiratory signs and symptoms, Sri Lanka

## Abstract

**Background:**

Worldwide, around 3 billion people rely on solid fuel for their daily energy needs. Household air pollution secondary to solid fuel burning is a major risk factor for respiratory mobility among vulnerable populations. This study aimed to investigate the respiratory symptoms associated with solid fuel usage, the level of kitchen fuel smoke exposure and its association with respiratory symptoms among reproductive-aged women in Sri Lanka, where most households exclusively use firewood as the primary cooking fuel.

**Methods:**

A descriptive cross-sectional study was conducted among 403 reproductive-aged women (15 to 49 years) in the Central Province, Sri Lanka. A structured interviewer-administered questionnaire was used to collect data, and an exposure assessment was done using a breath carbon monoxide monitor.

**Results:**

After adjusting for potential confounding factors by the logistic regression models, the odds ratios (OR) of the liquid petroleum gas-only users for at least one respiratory symptom relevant to cough (OR: 0.39; 95% confidence interval [CI]: 0.20–0.78), wheezing (OR: 0.47; 95% [CI]: 0.26–0.87), and dyspnea (OR: 0.44; 95% CI: 0.24–0.84) were significantly lower compared to firewood-only users. The mean of expired air carbon monoxide and estimated carboxyhemoglobin levels of liquid petroleum gas-only users (2.84 ± 2.85 ppm; 1.08 ± 0.46%) were significantly lower than those of firewood-only users (5.27 ± 4.64 ppm; 1.47 ± 0.74%).

**Conclusions:**

The use of firewood increased the risk of respiratory symptoms among reproductive-aged women in Sri Lanka. Health education focused on positive behavioral changes and effective and efficient clean energy policies are recommended to mitigate the risk associated with solid fuel smoke exposure.

## Introduction

"Solid fuel use (SFU)" refers to the household combustion of biomass fuels such as wood, cow dung, charcoal, or crop leftovers. Around 3 billion people worldwide, especially in low-and middle-income countries, continue to use solid fuels for cooking or heating purposes due to the lack of access to clean or contemporary energy sources [1]. Solid fuels are frequently burned in inefficient cooking stoves with inadequate ventilation. Incomplete combustion of solid fuel results in emissions of a large number of health-damaging pollutants, including respirable particles and carbon monoxide (CO), which significantly contribute to household air pollution (HAP). It is estimated that 3.8 million people of the global population will die prematurely due to this overlooked health danger in 2016 [2]. According to a recent study, exposure to daily HAP concentrations in women and young girls is typically seven times greater than in males and young boys [3]. This situation is more prevalent in South Asian countries, where women of reproductive age shoulder a disproportionate share of culinary responsibilities, exposing them to high levels of HAP daily.

Concurrently, solid fuel consumption is strongly linked with acute lower respiratory infections in young children, as well as chronic obstructive pulmonary disease and lung cancer in women [1, 4]. Furthermore, a global study involving 91 developing nations showed that the use of solid fuels is linked to a greater incidence of female mortality from indoor air pollution in underdeveloped nations [5]. Hence, household SFU is expected to be a substantial source of illness burden in the communities where it is prevalent. Nevertheless, the magnitude of the problem varies considerably across countries, and the evidence on respiratory symptoms and personal level of exposure has been limited. Thus, quantifying the national-level impact of SFU is critical for identifying and prioritizing environmental and public health interventions.

Sri Lanka is a South Asian country that has recently upgraded into a lower-middle-income country. In Sri Lanka, the consumption of solid fuels and the associated disease burden are pronounced. Over 66% of households in Sri Lanka use wood as a cooking fuel; 25%, 74%, and 80% of these households are in the urban, rural, and estate sectors, respectively [6]. However, research conducted in Sri Lanka found that particulate matter levels, especially PM_2.5_ levels, surpassed the World Health Organization's recommended guidelines in more than 70% of households utilizing wood as a cooking fuel [7]. In line with that, World Health Organization projected that 4300 people died in Sri Lanka due to HAP related to SFU in 2004 [8]. Conversely, this topic is under-researched and under-represented in Sri Lanka's public health policies and programs. Therefore, we investigated the impact of SFU on respiratory symptoms among reproductive-aged Sri Lankan women. We also examined the breath carbon monoxide and estimated carboxyhemoglobin levels as exposure assessments and their association with the respiratory symptoms.

## Methods

### Study design and sample

A cross-sectional study was conducted among 403 reproductive-aged women (15 to 49 years) in the Central Province of Sri Lanka. Central Province is the sixth-largest of Sri Lanka's nine provinces, with 2.5 million people. Kandy, Matale, and Nuwara-Eliya are the three districts that make up the province. Kandy district has 22 Medical Officer of Health (MoH) areas, whereas Matale and Nuwara-Eliya districts each have 13 MoH areas. MoH areas are clearly defined health units in the community that correspond to the country's administrative divisions, whereas Public Health Midwife (PHM) areas are the smallest operational units in the government healthcare system.

A multi-stage cluster survey was conducted in the Central Province, encompassing all three districts. A simple random sample technique was utilized to choose eight MoH areas from the Kandy district, four MoH areas from Matale district, and four MoH areas from Nuwara Eliya district for the first stage of sampling at the district level. Three PHM areas were chosen randomly from each selected MoH area for the second stage. The number of subjects from each district was determined using the probability proportional to size technique, considering the population resident in that district, based on the data from the fourteenth national Census conducted in 2012 [9]. The total number of participants in each district was then distributed nearly equally among selected MoH areas. According to the systematic selection, the researcher chose a random beginning point in each PHM area for data collection and then every second house after that. If a qualifying subject was not available in a selected house, choose the next closest house instead and continue the systematic selection process as usual. This was done until the required sample size was reached. Reproductive-aged women who had household cooking responsibilities and lived in Central Province, Sri Lanka, from January 2020 to December 2021 were included in the study. Women who were previously diagnosed with heart diseases, chronic chest-related diseases, asthma, and acute or chronic bronchitis were excluded from the study.

### Data collection

Data were collected using a pre-tested questionnaire. The questionnaire included questions on socio-demographic and economic data; use of cooking fuel; characteristics of the kitchen; exposure to other types of indoor air pollution such as active or passive tobacco smoke and mosquito coil smoke; and the prevalence of respiratory symptoms such as cough, phlegm, wheezing, and dyspnea. If a woman reported having respiratory symptoms most days of the year for five consecutive months or longer, she was considered to have the respiratory condition. Subsequently, expired air CO concentrations and percentages of COHb were measured using a breath CO monitor called the Micro + TM Smokerlyzer® (Bedfont Scientific Ltd., Maidstone, UK) within three hours of meal preparation, either in the morning or in the afternoon. Women were instructed to hold their breath for 15 s before blowing into the instrument. CO in the breath was quantified in parts per million (ppm), and estimated COHb levels were quantified as the percentage of oxygen replaced. The breath CO monitor is accurate within ± 2 ppm, and it was calibrated at least every six months, as recommended by the manufacturer. CO levels were classified as equivalent to non-smokers (0–6 ppm), borderlines smokers (7–9 ppm), low-addicted smokers (10–15 ppm), moderately addicted smokers (16–25 ppm), and heavily addicted smoker (> 26 ppm). The corresponding COHb levels were defined as 0.00–1.59, 1.75–2.23, 2.39–3.03, 3.19–4.63, and > 4.79%, respectively [10]. Because breath CO is not a precise biomarker of smoking but it is also impacted by environmental factors such as motor vehicle emissions, women exposed to motor vehicle fumes within three hours of data collection were not recruited into the study.

### Data management and data analysis

Data analyses were done using the Minitab 20 statistical software. Descriptive statistics are shown as means and standard deviations (SD) or as frequencies and percentages. All continuous variables were first evaluated numerically and graphically (e.g., scatter plots) to ensure that they satisfied the distributional assumption of the statistical tests employed in their analysis. Two-sample proportion test was used to compare respiratory symptoms among users of different types of cooking fuel. And two-sample t-test was used to compare the mean CO and COHb levels based on the presence and absence of respiratory symptoms. The factors related to respiratory symptoms in reproductive-aged women were identified using univariate and multivariate binary logistic regression analysis. We conducted univariate and multivariate analyses using the selected respiratory symptom as the dependent variable and age, civil status, level of education, employability, monthly family income, family structure, type of cooking fuel, duration of cooking, the status of tobacco smoke exposure, the status of exposure to mosquito coil smoke, and kitchen characteristics as independent variables. At first, univariate analyses were performed to determine the associations between the hypothesized independent variables and the presence of cough, phlegm, wheezing, and dyspnea. When modeling the multivariate analysis for the presence of cough, women who reported at least one of the studied cough-related symptoms were considered to have a cough. A similar procedure was used for phlegm, wheezing, and dyspnea models. Then, for the multivariate binary logistic regression models, variables having a p-value less than or equal to 0.3 in the univariate analyses were chosen. Multicollinearity was determined by performing a linear regression analysis, keeping one of the independent variables as the dependent variable and the remaining independent variables as the independent variables. The variance inflation factor (VIF) was used to determine multicollinearity, and variables with a VIF < 5 were subsequently included in multivariate logistic regression to find factors associated with respiratory symptoms. The odds ratio (OR) and corresponding 95% confidence interval (CI) were calculated to determine the statistical significance of the related variables. The one-way analysis of variance (ANOVA) was performed to assess the differences in the means of exhaled breath CO levels and estimated COHb percentages based on the women's most recently utilized cooking fuel. A multivariate model was created to describe the independent variables affecting the CO level in exhaled breath while controlling for confounding variables. 95% CIs were generated for each analysis, and *p* < 0.05 was considered statistically significant.

## Results

### Characteristics of the study participants

The study enrolled 403 women of reproductive age, including 50.4% from Kandy and 24.8% from Matale and Nuwara-Eliya districts. The participants' average age was 37.4 ± 8.9 years. The average family size was four members per household (Table 1).

**Table 1:** Demographic characteristics of study participants

Variable	Frequency (%)	Mean ± SD
Age (years)		37.4 ± 8.9
Civil status		
Single	58 (14.4)	
Married	318 (78.9)	
Divorced	13 (3.2)	
Widowed	14 (3.5)	
Level of education		
No school education	6 (1.5)	
Up to primary	43 (10.7)	
Up to ordinary level	184 (45.6)	
Up to advanced level	124 (30.8)	
Higher education	46 (11.4)	
Employability		
Employed	170 (42.2)	
Unemployed	233 (57.8)	
Monthly family income (LKR)		
< 14,999	15 (3.7)	
15,000–22,499	59 (14.6)	
22,500–45,999	173 (42.9)	
46,000–149,999	155 (38.5)	
> 150,000	1 (0.3)	
Type of the Family		
Nuclear	290 (72.0)	
Extended	113 (28.0)	
Average number of people per household		4.3 ± 1.6
Average number of males per household		2.1 ± 1.0
Average number of females per household		2.2 ± 1.1

Continuous variables expressed by the mean and standard deviation (SD); categorical variables described by number and frequencies and percentages

*Abbreviation: LKR* Sri Lankan rupee

### Cooking fuel usage and characteristics of the kitchen

23.1% of households in the entire sample used only firewood as their primary source of cooking fuel, while 28% used liquid petroleum gas (LPG) exclusively. Nevertheless, 48.6% of respondents reported using firewood in conjunction with LPG or kerosene, increasing the overall percentage of firewood users to 71.1%. Almost every household (98.7%) cooks inside the houses, often in a separate kitchen. In houses that cook with firewood, 79.9% and 91% of kitchens, respectively, were designed with a kitchen chimney and windows. Even though most residences were designed with windows, only 71.9% reported opening windows when cooking with firewood. 95.8% of the total sample was used to cook daily, spending an average of 158 ± 80.6 min every day (Table 2).

**Table 2:** Household cooking activities and the characteristics of the kitchen

Variable	Frequency (%)
Type of kitchen fuel	
Firewood only	93 (23.1)
LPG only	113 (28.0)
Kerosene only	01 (0.3)
Firewood plus LPG	179 (44.4)
Firewood plus kerosene	17 (4.2)
Characteristics of the kitchen^a^	
Having a chimney	231 (79.9)
Having windows	263 (91.0)
Open the windows during cooking time^b^	189 (71.9)
Having ventilation holes	190 (65.7)
Frequency of cooking	
Daily	386 (95.8)
Few days in a week	17 (4.2)
Average time taken for cooking activities (in minutes) ^c^	158 ± 80.6
Method of starting a fire when using firewood ^a^	
Using a pipe	154 (53.3)
Using exhaled breath	104 (36.0)
Direct firing of the firewood without using exhaled breath	31 (10.7)

*Abbreviations: LPG* liquid petroleum gas

^a^Considered only the people who used firewood as a kitchen fuel; *n* = 289

^b^Out of the households with windows in the kitchen; ^c^ for the daily cooking participants only and expressed as mean ± SD

### Exposure to additional sources of household air pollution

No women reported being active smokers; however, 35.7% were exposed to secondhand tobacco smoke. 45.1% of the women exposed to tobacco smoke were exposed in their houses, while 18.5% reported daily exposures inside their houses. More than half of the total secondhand tobacco smoke cases (55.5%) were women who had been exposed while their husbands were smoking. 22.1% of women reported using mosquito coils at night most days, inside their houses.

### Comparison of respiratory symptoms among different types of cooking fuel users

Coughing usually and coughing on getting up were significantly higher in firewood-only users and in firewood alone or in combination users than in LPG-only users (*p* < 0.05). When comparing firewood plus LPG users to LPG-only users, the prevalence of bringing up phlegm from the chest and bringing up phlegm at all on getting up was significantly lower among LPG-only users (*p* < 0.05). Significantly more firewood-only users than LPG-only users reported whistling sounds in their chest with cold. Additionally, when compared to LPG-only users, those who used firewood alone or in combination walked slower than people at their age due to shortness of breath and pausing for breath while walking at their own pace on level (p < 0.05) (Table 3).

**Table 3:** Prevalence of respiratory symptoms based on the cooking fuel type

Respiratory symptom	Firewood only users [*n* = 93] n (%)	LPG-only users [*n* = 113] n (%)	Kerosene only users [*n* = 01] n (%)	Firewood + LPG users [*n* = 179] n (%)	Firewood + Kerosene users [*n* = 17] n (%)	Firewood alone or in combination users [*s* = 289] n (%)
Cough						
Having cough usually	31 (7.6)^a,*^	17 (4.2)	01 (0.2)	36 (8.9)	06 (1.5)	73 (18.1)^c, *^
Having cough 4–6 times a day, four or more days out of the week	6 (1.5)	6 (1.5)	00 (0.0)	13 (3.2)	01 (0.2)	20 (5.0)
Cough at all on getting up	13 (3.2)^a, *^	5 (1.2)	00 (0.0)	24 (6.0)	02 (0.5)	39 (9.7)^c, *^
Cough at all during the rest of the day or night	13 (3.2)	10 (2.5)	01 (0.2)	09 (2.2)	03 (0.7)	25 (6.2)
Phlegm						
Bringing up phlegm from the chest usually	38 (9.4)	34 (8.4)	01 (0.2)	84 (20.8)^b, *^	08 (2.0)	130 (32.3)^c, *^
Bringing up phlegm twice a day, four or more days out of the week	15 (3.7)	15 (3.7)	00 (0.0)	39 (9.7)	06 (1.5)	60 (14.9)
Bringing up phlegm at all on getting up	23 (5.7)	17 (4.2)	01 (0.2)	54 (13.4)^b, *^	05 (1.2)	82 (20.3)
Bringing up phlegm at all during the rest of the day or at night	16 (4.0)	17 (4.2)	00 (0.0)	29 (7.2)	04 (1.0)	49 (12.2)
Wheezing						
Having whistling sound on the chest with cold	34 (8.4)^a, *^	22 (5.5)	01 (0.2)	36 (8.9)	08 (2.0)	78 (19.3)
Having whistling sound on chest without cold	15 (3.7)	10 (2.5)	01 (0.2)	18 (4.5)	02 (0.5)	35 (8.7)
Having whistling sound on chest at most nights	05 (1.2)	09 (2.2)	01 (0.2)	13 (3.2)	01 (0.2)	19 (4.7)
Experienced of wheezing attacks that caused short of breath	25 (6.3)	19 (4.8)	01 (0.2)	28 (7.0)	06 (1.5)	59 (14.6)
Treated with medicine for wheezing attacks	20 (5.0)	17 (4.2)	01 (0.2)	27 (6.7)	07 (1.7)	54 (13.4)
Dyspnea						
Troubled by dyspnea when hurrying in the level or walking up slight hill	29 (7.2)	24 (6.0)	00 (0.0)	38 (9.4)	08 (2.0)	75 (18.6)
Slower than people of own age on level because of breathlessness	17 (4.2)^a^	09 (2.2)	00 (0.0)	25 (6.2)	02 (0.5)	44 (10.9)^c^
Stop for breath when walking at the own pace on the level	18 (4.5)^a^	09 (2.2)	00 (0.0)	38 (9.4)^b^	03 (0.7)	59 (14.6)^c^

*Abbreviation: LPG* Liquid petroleum gas

^a^Significant difference was identified when comparing firewood only users and LPG-only users

^b^Significant difference was identified when comparing LPG only users and firewood plus LPG users

^c^Significant difference was identified when comparing LPG only users and firewood alone or in combination users

^*^p < 0.05

### Effect of cooking fuel use on respiratory symptoms

By controlling for the effect of other variables, multivariate logistic regression analyses were utilized to determine the presence of an association between cooking fuel type and respiratory symptoms. Four distinct multivariate logistic regression models were built for cough, phlegm, wheezing, and dyspnea.

The fitted multivariate logistic regression model for cough-related respiratory symptoms revealed that women who cook only with firewood have a higher risk of cough-related symptoms than women who cook only with LPG (OR:0.39; 95% confidence interval [CI]: 0.20–0.78) or women who cook with firewood plus LPG (OR:0.54; 95% CI: 0.30–0.96) (*p* < 0.05). Simultaneously, it was found that women exposed to secondhand tobacco smoke are more likely to experience cough-related respiratory symptoms than women that are not exposed (OR:0.58; 95% CI: 0.35–0.96) (*p* < 0.05) (Table 4). No significant effect was found on phlegm-related respiratory symptoms among the participants relevant to the type of cooking fuel (Table 5). In line with the findings for cough-related symptoms, the model fitted for wheezing-related symptoms indicated that LPG-only users (OR: 0.47; 95% [CI]: 0.26–0.87) and firewood plus LPG users (OR:0.49; 95% CI: 0.50–4.35) are at a lower risk of having wheezing-related symptoms than firewood-only users. Additionally, it was noted that secondhand tobacco smoke was a risk factor for wheeze in reproductive-aged women (*p* < 0.001) (Table 6). Dyspnea symptoms were less likely to occur in LPG-only users (OR: 0.44; 95% CI: 0.24–0.84) compared to firewood-only users (*p* < 0.05) (Table 7).

**Table 4:** Multivariate analysis for the presence of at least one cough-related symptom

Variable in model	Number of subjects (*n* = 402)		Odds Ratio	95% CI	*P* value
	**Presence of symptoms** **(n = 95)**	**Absence of symptoms** **(n = 307)**			
Age			1.03	0.10–1.06	0.081
Civil status					
Single (Reference level)	14	44			
Married	77	240	0.77	0.38–1.60	0.488
Divorced	01	12	0.16	0.02–1.41	0.098
Widowed	03	11	0.61	0.14–2.72	0.515
Type of cooking fuel^a^					
Firewood only (Reference level)	31	62			
LPG only	18	95	0.39	0.20–0.78	0.007^*^
Firewood plus LPG	40	139	0.54	0.30–0.96	0.034^*^
Firewood plus kerosene	06	11	1.04	0.34–3.25	0.945
Exposure to second-hand tobacco smoking					
Yes (Reference level)	42	102			
No	53	205	0.58	0.35–0.96	0.033^*^
Having kitchen windows					
Yes	89	271			
No (Reference level)	06	36	0.51	0.20–1.29	0.154

*Abbreviations: CI* confidence interval, *LPG* liquid petroleum gas

^a^Kerosene only category was excluded from the analysis as there was only one subject in that particular category

^*^p < 0.05

**Table 5:** Multivariate analysis for the presence of at least one phlegm-related symptom

Variable in model	Number of subjects (*n* = 401)		Odds Ratio	95% CI	*P* value
	**Presence of symptoms** **(*n* = 170)**	**Absence of symptoms** **(*n* = 231)**			
Age			1.03	0.10–1.05	0.045^*^
Civil status					
Single (Reference level)	23	35			
Married	140	176	0.98	0.52–1.86	0.947
Divorced	03	10	0.33	0.07–1.46	0.143
Widowed	04	10	0.37	0.09–1.33	0.155
Employability					
Unemployed (Reference level)	95	137			
Employed	75	94	1.34	0.87–2.06	0.182
Type of cooking fuel^a^					
Firewood only(Reference level)	40	53			
LPG only	34	79	0.62	0.34–1.12	0.112
Firewood plus LPG	87	91	1.23	0.73–2.08	0.434
Firewood plus kerosene	09	08	1.77	0.60–5.24	0.306
Having kitchen windows					
Yes (Reference level)	158	202			
No	12	29	0.41	0.18–0.92	0.031^*^
Keep the kitchen windows open while cooking					
Yes (Reference level)	107	159			
No	63	72	1.88	1.15–3.08	0.012^*^

*Abbreviations: CI* confidence interval, *LPG* liquid petroleum gas

^a^Kerosene only category was excluded from the analysis as there was only one subject in that particular category

^*^p < 0.05

**Table 6:** Multivariate analysis for the presence of at least one wheezing-related symptom

Variable in model	Number of subjects (*n* = 402)		Odds Ratio	95% CI	*P* value
	**Presence of symptoms** **(*n* = 117)**	**Absence of symptoms** **(*n* = 285)**			
Family type					
Nuclear (Reference level)	88	202			
Extended	29	83	0.66	0.39–1.11	0.120
Type of cooking fuel^a^					
Firewood only (Reference level)	37	56			
LPG only	28	85	0.47	0.26–0.87	0.016^*^
Firewood plus LPG	43	136	0.49	0.28–0.85	0.012^*^
Firewood plus kerosene	09	08	1.47	0.50–4.35	0.484
Having kitchen windows					
Yes	101	259			
No (Reference level)	16	26	1.54	0.77–3.08	0.226
Exposure to second-hand tobacco smoking					
Yes (Reference level)	58	86			
No	59	199	0.42	0.26–0.68	< 0.001^*^
Exposure mosquito coil smoke at night					
Yes (Reference level)	25	64			
No	92	221	1.63	0.91–2.90	0.101

*Abbreviations: CI* confidence interval, *LPG* liquid petroleum gas

^a^Kerosene only category was excluded from the analysis as there was only one subject in that particular category

^*^p < 0.05

**Table 7:** Multivariate analysis for the presence of at least one dyspnea-related symptom

Variable in model	Number of subjects (*n* = 402)		Odds Ratio	95% CI	*P* value
	**Presence of symptoms** **(*n* = 119)**	**Absence of symptoms** **(*n* = 283)**			
Age			1.03	1.01–1.06	0.017^*^
Employability					
Unemployed (Reference level)	64	169			
Employed	55	114			0.112
Type of cooking fuel^a^					
Firewood only (Reference level)	35	58			
LPG only	24	89	0.44	0.24–0.84	0.012^*^
Firewood plus LPG	52	127	0.63	0.37–1.09	0.096
Firewood plus kerosene	08	09	1.75	0.59–5.25	0.315
Exposure to second-hand tobacco smoking					
Yes (Reference level)	46	98			
No	73	185	0.75	0.46–1.22	0.248
Exposure mosquito coil smoke at night					
Yes (Reference level)	21	68			
No	98	215	1.73	0.95–3.14	0.073

*Abbreviations: CI* confidence interval, *LPG* liquid petroleum gas

^a^Kerosene only category was excluded from the analysis as there was only one subject in that particular category; ^*^p < 0.05

### Exposure–response assessment

Exhaled breath CO measurements revealed that 83.1% (*n* = 335) were at the non-smoker level, while 8.5% (*n* = 34) were at the borderline smoker level. Similar percentages (4.2%) of women were identified as in low-addicted smoker level and moderately addicted smoker level.

**Fig. 1:**
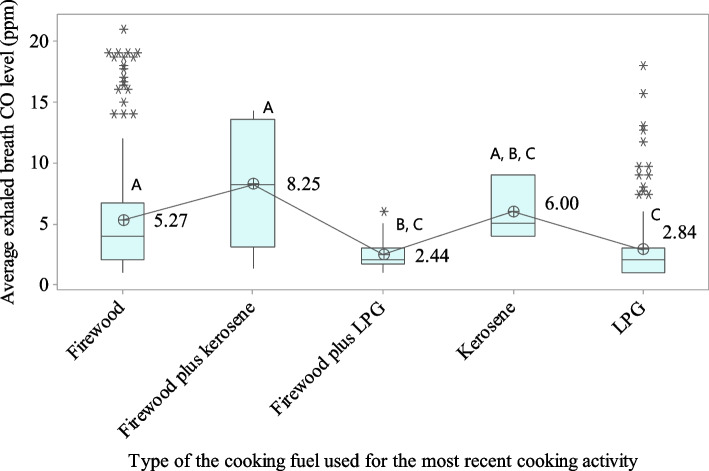
Box plot of mean exhaled air CO concentrations based on the most recently used cooking fuel type. ^A, B, C^Values with same uppercase letter do not represent significant mean difference; Abbreviation: CO = carbon monoxide, LPG = liquid petroleum gas

**Fig. 2:**
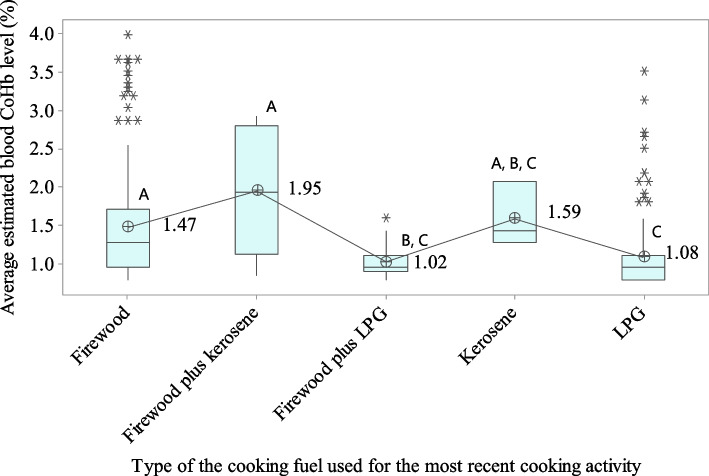
Box plot of mean estimated COHb percentages based on the most recently used cooking fuel type. ^A, B, C^Values with same uppercase letter do not represent significant mean difference; Abbreviation: COHb = carboxyhemoglobin, LPG = liquid petroleum gas

The overall mean concentration of CO in expired breath and estimated COHb percentage were 4.09 ± 4.0 ppm and 1.29 ± 0.64, respectively. The CO and COHb levels in exhaled breath were compared based on the type of cooking fuel used by the women in their most recent cooking activity within three hours of the measurement. 48.1% of women used only firewood for their most recent cooking activity, whereas 37.5%, 2.0%, 11.7%, and 0.7%, respectively, used only LPG, firewood plus kerosene, firewood plus LPG, and only kerosene. The univariate analysis revealed that LPG-only users had considerably lower exhaled breath CO levels and estimated COHb percentages than firewood-only users and firewood plus kerosene users (Figs. 1 and 2).

The fitted multivariate model (R^2^ (adjusted) = 36.84%) indicated that the duration of cooking or the time spent exposed to cooking fuel smoke, civil status, employability, the type of cooking fuel used in the most recent cooking activity, and exposure to secondhand tobacco smoke all had a significant effect on exhaled breath CO levels (*p *< 0.05).

The mean CO level in the expired air of women who cooked only with LPG was 1.98 ppm lower than the CO level in women who cooked only with firewood (*p* < 0.001). Similarly, consumers of firewood and LPG had a 1.62 ppm lower breath CO level than users of firewood alone (*p* = 0.003). Significantly, it was found that women exposed to secondhand tobacco smoke had an exhaled CO level of 2.74 ppm higher than the non-exposed comparator (*p* < 0.001) (Table 8).

**Table 8:** Multivariate regression model for exhaled breath CO level (ppm): *R*^2^ (adjusted) = 36.84%, *n* = 367

Variable in model	Coefficient	95% CI	t	*p*-Value
Constant	5.20	2.97–7.43	4.59	< 0.001^*^
Duration of cooking (minutes per day)	0.005	0.001–0.010	2.43	0.016*
Civil status				
Single (Reference level)				
Married	0.85	–0.11–1.87	1.74	0.082
Divorced	3.59	1.17–6.01	2.92	0.004^*^
Widowed	2.02	–0.21–4.25	1.79	0.075
Employability				
Unemployed (Reference level)				
Employed	1.17	0.47–1.86	3.30	0.001^*^
Monthly family income (LKR)				
< 14,999 (Reference level)				
15,000–22,499	0.95	–1.17–3.07	0.88	0.378
22,500–45,999	–0.55	–2.53–1.44	–0.54	0.588
46,000–149,999	–1.60	–3.63–0.43	–1.55	0.122
> 150,000	–1.82	–8.42–4.78	–0.54	0.588
Type of the cooking fuel used for the most recent cooking activity^a^				
Only firewood only (Reference level)				
Only LPG	–1.98	–2.76– –1.19	–4.96	< 0.001^*^
Only kerosene	1.89	–1.78–5.57	1.01	0.312
Firewood plus LPG	–1.62	–2.67– –0.57	–3.04	0.003^*^
Firewood plus kerosene	0.84	–1.45–3.14	0.72	0.471
Having kitchen windows				
Yes (Reference level)				
No	–1.19	–2.50–0.11	–1.80	0.072
Keep the kitchen windows open while cooking				
Yes (Reference level)				
No	0.69	–0.14–1.52	1.62	0.105
Exposure to second-hand tobacco smoking				
Yes (Reference level)				
No	–2.74	–3.53– –1.95	–6.84	< 0.001^*^

*Abbreviations: CI* confidence interval, *LKR* Sri Lankan rupee, *LPG* liquid Petroleum gas

^a^Within three hours of the measurement; *p < 0.05

Women who reported at least one respiratory symptom relevant to cough, phlegm, wheezing and dyspnea had significantly higher mean expired air CO concentrations and estimated COHb percentages compared to the negative comparator (*p* < 0.05) (Table 9).

**Table 9:** Breath CO and COHb levels based on the presence or absence of respiratory symptoms, *n* = 402

Respiratory symptom	Expired air CO concentration (ppm)^a^					Estimated COHb percentage (%)^a^				
	**Presence of respiratory symptom**		**CI for difference**	**t**	***p*-value**	**Presence of respiratory symptom**		**CI for difference**	**t**	***p*-value**
	**Yes (n)**	**No (n)**				**Yes (n)**	**No (n**			
Presence of at least one cough-related symptom	5.18 ± 4.79 (96)	3.75 ± 3.66 (307)	0.37–2.47	2.67	0.008^*^	1.46 ± 0.77 (96)	1.23 ± 0.59 (307)	0.06–0.40	2.72	0.007^*^
Presence of at least one phlegm-related symptom	4.68 ± 4.39 (171)	3.67 ± 3.65 (231)	0.20–1.82	2.45	0.015^*^	1.38 ± 0.70 (171)	1.22 ± 0.58 (231)	0.03–0.29	2.49	0.013^*^
Presence of at least one wheezing-related symptom	5.22 ± 4.39 (118)	3.63 ± 3.74 (285)	0.68–2.50	3.45	0.001^*^	1.47 ± 0.70 (118)	1.21 ± 0.60 (285)	0.11–0.40	3.49	0.001^*^
Presence of at least one dyspnea-related symptom	4.92 ± 4.48 (119)	3.75 ± 3.74 (284)	0.25–2.10	2.52	0.013^*^	1.42 ± 0.72 (119)	1.23 ± 0.60 (284)	0.40–0.34	2.51	0.013^*^

*Abbreviations: CO* carbon monoxide, *COHb* carboxyhemoglobin, *CI* confidence interval

^a^Mean ± SD; ^*^p < 0.05

## Discussion

Although our findings are limited to one province in Sri Lanka, this is the first study that we are aware of examining respiratory symptoms and personal CO levels in reproductive-aged women in Sri Lanka. This cross-sectional study mainly aimed to determine the effect of SFU on respiratory symptoms in reproductive-aged women living in Central Province, Sri Lanka. According to the current study, 23.1% of households used firewood exclusively, while 48.6% used firewood in conjunction with LPG or kerosene, bringing the total percentage of families that used firewood to 71.1%. This percentage is greater than the national data for the use of firewood as a cooking fuel (66%) reported in 2016 [6]. This higher percentage may be attributable to the abundance of firewood in Central Province compared to the countryside and the cost-effectiveness compared to LPG.

We discovered a higher prevalence of respiratory symptoms among firewood users, which is similar to the findings from Nepal [11], Bangladesh [12], and Mexico [13]. However, a study conducted in Nigeria found a low prevalence of coughing, wheezing, and dyspnea in both the biomass and non-biomass groups [14], which could be attributed to underreporting of respiratory symptoms that were frequently regarded as normal by the general population [11]. Except for phlegm, our findings support a higher incidence of cough-related symptoms, wheezing, and dyspnea among firewood users alone or in conjunction with LPG. These relationships persisted even after controlling for a variety of significant confounding variables.

Various contaminants, particularly respirable particulate matter, contribute to respiratory symptoms [15]. CO is regarded as a reliable proxy for individual exposure to respirable particles [16]. The current study found that women who reported respiratory symptoms had significantly higher CO and COHb levels. Moreover, firewood-only users had significantly higher mean CO and COHb levels than LPG-only users, implying that CO concentration may be a significant determinant of respiratory symptoms. In line with current study findings, a study carried out in rural Uganda revealed a connection between personal CO levels and exposure to biomass smoke and, consequently, a higher risk of respiratory symptoms in women who use biomass cooking fuels [17].

In Sri Lanka, the majority of cooking tasks are performed by women, while young children and elderly family members remain indoors, implying that a sizable portion of the population is exposed to HAP created by firewood burner operation [18]. Despite the fact that no research has been published in Sri Lanka that examined the respiratory health of reproductive-aged women and their personal CO levels, a study among children in a semi-urban Sri Lankan community found considerably higher CO and PM_2.5_ concentrations in the indoor air of households using wood fuel and a 1.6-fold increased risk of infection-induced asthma in children in high exposure groups [19]. Partially dried firewood is difficult to light and produces a lot of smoke. As a result, it is common to utilize a fire starter made from dried coconut leaves or waste papers, which results in much smoke being released during the fire-starting process. In addition, most women practiced using forcibly exhaled air to assist in starting fires, putting them at risk of breathing in excessive amounts of firewood smoke. According to the current study, 36% of firewood users reported using forced exhaled breath to start a fire, while 53% used a pipe to supply exhaled breath air to start a fire, both of which are extremely harmful behaviors that continue to be done due to a lack of understanding. Nonetheless, Sri Lankan women tend to stay close to the cooking stoves throughout the cooking process, which might cause harm even at modest levels of exposure over time. The current analysis found that firewood users spend an average of 158 ± 80.6 min per day cooking.

Studies suggest that well-designed and well-ventilated kitchen structures could help to limit exposure to cooking fuel smoke. According to the current study, 79.9% of firewood users had kitchens with chimneys. However, no significant difference in CO levels was found between women who had kitchen chimneys and women who did not have kitchen chimneys (data not shown). This could be because some kitchens have doors, kitchen windows, and existing open ventilation holes. Unfortunately, only 71.9% of households with windows in the kitchen practiced opening the windows while cooking with firewood.

While adjusting for other factors, the current study found that secondhand tobacco smoke was a risk factor for wheezing in reproductive-aged women (p < 0.001). The average CO concentration in this study was 4.09 ± 4.0 ppm, which was higher than the average CO levels reported for healthy non-smokers in a Turkish study [20], but lower than the levels seen among solid fuel users in Guatemala [21]. Furthermore, 8.5%of the women in this study sample had CO levels that fell into the borderline smoker category (7–9 ppm), while 4.2 percent fell into the low-addicted (10–15 ppm) and moderately addicted (16–25 ppm) categories, respectively. The problem is that these women had never smoked and had accidentally fallen into these risk categories due to this unaddressed community health concern. Thus, while tobacco smoking is a major cause of respiratory morbidity in the developed world, HAP exposure is expected to be a significant modifiable risk factor in low- and middle-income countries, particularly among women [22].

The current study focused specifically on reproductive-aged women, with a reported mean age of 37.4 ± 8.9 years. Firewood smoke exposure has a detrimental effect on not just their respiratory health but also on their reproductive health. The adverse perinatal outcomes associated with biomass fuel smoke exposure in pregnant women are well documented and include but are not limited to low-birth-weight deliveries [23–25], preterm births [23–25], stillbirths [24, 25] and neonatal mortality [25, 26]. Thus, this research area addresses a timely and significant health issue that can negatively impact both present and future generations, both directly and indirectly. Improved cooking stoves, alternate fuel sources, a better living environment, and changing user habits to decrease exposure have been suggested to decrease firewood smoke exposure among vulnerable populations [27]. Pre-processing (drying) the fuel, proper stove maintenance, and installing stoves at waist level are reasonable and affordable options in the Sri Lankan context to reduce the cooking fuel smoke exposure where most people belong to the middle-income level. Simple changes in user behavior to decrease their exposure to kitchen fuel smoke include utilizing a pot lid to preserve the heat while cooking, pre-soaking foods like grains and dhal to reduce cooking time, and not using the exhaled breath to start a fire in a wood stove [27]. However, the expansion of LPG is hampered significantly by its high price, limited supply and access in the Sri Lankan context; therefore, policymakers could supply cost-effective, efficient stoves to help reduce smoke exposure in Sri Lanka's low-resource areas.

To our knowledge, this is the first study to examine household characteristics, cooking behaviors, respiratory symptoms, and CO and COHb levels in expired breath among reproductive-aged women in a Sri Lankan community. This study adds to a growing body of knowledge about the impact of HAP on respiratory symptoms and personal CO levels. This study relies on self-reported cooking habits and objective data on personal exposure levels. There were a few limitations, despite the study's strengths. Some parts of the Central Province have a cold climate, which may have influenced our findings regarding respiratory symptoms. Also, the sample size used in this study may not be adequate to compensate for the bias introduced by the cluster sampling technique. Furthermore, we did not look at how long women used different types of cooking fuel over the years.

## Conclusion

This study indicates an increased risk of respiratory symptoms among firewood cooking fuel users. Health education for wood fuel users, focused on positive behavioral changes, is strongly recommended. The findings of this study may have implications for both research and public policy development aimed at improving respiratory health by minimizing exposure to solid fuel smoke and increasing access to contemporary clean fuel types. Further interventional studies are recommended to address the effects of different sources of HAP and to enhance respiratory health among reproductive-aged women.

## Data Availability

The data that support the findings of this study are available on request from the corresponding author.
